# Hormonal Profile Abnormalities Among Oligozoospermic and Azoospermic Men With Infertility: A Cross-Sectional Study

**DOI:** 10.7759/cureus.116391

**Published:** 2026-09-16

**Authors:** Rukhsana Tumrani, Afsheen Nigar, Ali Imran, Syed Atif Hussain, Farhana Ashraf, Tamia Khan, Muhammad Usman, Amna Rehman, Warda Saeed, RizwanUllah Hamdard

**Affiliations:** 1 Chemical Pathology, King Edward Medical University, Lahore, PAK; 2 Chemical Pathology, University College of Medicine and Dentistry, The University of Lahore, Lahore, PAK; 3 Pediatric Surgery, Sindh Institute of Urology and Transplantation, Karachi, PAK; 4 Urology, Sheikh Zayed Medical College and Hospital, Rahim Yar Khan, PAK; 5 Pathology, Services Institute of Medical Sciences, Lahore, PAK; 6 Chemical Pathology, Bahawal Victoria Hospital, Quaid-e-Azam Medical College (QAMC), Bahawalpur, PAK; 7 Cardiology, Punjab Institute of Cardiology, Lahore, PAK; 8 Pathology, University College of Medicine and Dentistry, The University of Lahore, Lahore, PAK; 9 Microbiology, Institute of Molecular Biology and Biotechnology, The University of Lahore, Lahore, PAK

**Keywords:** azoospermia, hormonal imbalance, hormonal profile, hyperprolactinemia, male infertility, oligozoospermia, partial androgen resistance, primary hypergonadotropic hypogonadism, primary spermatogenic failure, secondary hypogonadism

## Abstract

Introduction

Male infertility contributes substantially to infertility worldwide, and hormonal abnormalities play an important role in impaired spermatogenesis and reproductive function. Evaluation of hormonal status may aid in identifying potentially treatable causes of male infertility.

Objective

This study aims to compare the frequency and pattern of abnormalities in serum follicle-stimulating hormone (FSH), luteinizing hormone (LH), testosterone, and prolactin between infertile men with oligozoospermia and those with azoospermia presenting to a tertiary care hospital.

Methods

This cross-sectional study was conducted in the Departments of Pathology and Urology at Sheikh Zayed Hospital, Rahim Yar Khan, Pakistan, from January 2021 to March 2022. Males aged 20-55 years with sperm concentration <15 million/mL on semen analysis (oligozoospermia) or absence of sperm in ejaculate (azoospermia), presenting for infertility evaluation and management, were included. Serum LH, FSH, testosterone, and prolactin levels were measured. Patients aged >55 years or with a history of chemotherapy, pelvic surgery, mumps, or hernia were excluded. Hormonal profiles were compared between oligozoospermic and azoospermic groups.

Results

Among 213 infertile males, the mean age was 34 ± 7.87 years. Oligozoospermia was present in 123 (57.7%) participants and azoospermia in 90 (42.3%). Among azoospermic males, seven (7.8%) had primary hypergonadotropic hypogonadism, 19 (21.1%) had secondary hypogonadism, nine (10.0%) had primary spermatogenic failure, four (4.4%) had a biochemical profile consistent with partial androgen resistance, three (3.3%) had hyperprolactinemia, and 48 (53.3%) had a normal hormonal profile. Corresponding frequencies among oligozoospermic males were four (3.3%), 24 (19.5%), 11 (8.9%), five (4.1%), two (1.6%), and 77 (62.6%), respectively. The overall distribution of hormonal-profile categories did not differ significantly between the oligozoospermic and azoospermic groups (exact P=0.593).

Conclusion

Hormonal abnormalities were observed in both oligozoospermic and azoospermic infertile men, although the distribution of the evaluated hormonal-profile categories did not differ significantly between the two groups. These findings support the clinical value of hormonal assessment as part of the etiological evaluation of selected men presenting with infertility.

## Introduction

Infertility is commonly defined as the inability of a couple to conceive after one year of regular, unprotected intercourse. Approximately 20% of infertility cases are attributable solely to male factors, while an additional 30%-40% involve male factors as contributory causes [1]. Overall, male factors account for nearly half of all infertility cases [2]. Therefore, comprehensive evaluation of male partners is essential to identify and manage underlying medical conditions that may contribute to infertility [3].

Male infertility may result from a wide range of etiologies, including hormonal, anatomical, genetic, metabolic, and idiopathic causes. The burden of infertility is generally higher in developing countries due to limited access to quality healthcare, high treatment costs, and social and cultural barriers. In resource-limited settings, diagnosis and management of infertility remain particularly challenging [4].

Semen abnormalities are considered a major contributor to male infertility; however, several additional etiological factors have also been recognized [5]. Semen analysis serves as the initial diagnostic tool, while endocrine evaluation plays an important role in selected patients. Measurement of gonadotropins and testosterone provides insight into the functional status of the hypothalamic-pituitary-testicular axis and assists in identifying hypogonadism in men presenting with oligozoospermia or azoospermia. In cases of suspected central hypogonadism, assessment of thyroid, adrenal, and lactotroph function is also recommended [5,6].

Men with reduced sperm counts are reported to have a substantially higher likelihood of hypogonadism and low testosterone levels [6,7]. Clinical assessment should include evaluation for endocrinopathies, gynecomastia, body hair distribution, and secondary sexual characteristics. Endocrine screening commonly includes measurement of serum testosterone, follicle-stimulating hormone (FSH), and luteinizing hormone (LH) [2,8].

Global trends indicate a progressive decline in sperm counts over recent decades. Although the exact causes remain uncertain, potential contributing factors include environmental exposures, lifestyle changes, and increasing recognition and diagnosis of reproductive disorders. Male infertility has also been associated with a greater burden of systemic comorbidities compared with fertile men [9].

The present study aimed to compare the frequency and pattern of abnormalities in serum FSH, LH, testosterone, and prolactin between infertile men with oligozoospermia and those with azoospermia presenting to a tertiary care hospital.

## Materials and methods

Study design

This cross-sectional study was conducted in the Departments of Pathology and Urology at Sheikh Zayed Hospital, Rahim Yar Khan, Pakistan, from January 2021 to March 2022. A non-probability consecutive sampling technique was used. A total of 213 eligible participants fulfilling the study criteria were included during the study period.

Study participants

Male patients aged 20-55 years presenting for infertility evaluation and management were included.

Semen Analysis

Semen samples were collected in the hospital laboratory by masturbation into a sterile, wide-mouthed container after seven days of sexual abstinence. Samples were analyzed within one hour of collection. Semen analysis was performed on a single sample from each participant according to the World Health Organization (WHO) 2010 laboratory manual. Oligozoospermia was defined as a sperm concentration of <15 million/mL according to the predefined study criterion. Azoospermia was defined as the absence of spermatozoa in the ejaculate, with confirmation by centrifugation and examination of the resulting pellet/sediment.

Patients older than 55 years or those with a history of chemotherapy, pelvic surgery, mumps, hernia, or non-residency in Pakistan were excluded.

Hormonal Profile Classification

Hormonal concentrations were classified as low, normal, or high according to the laboratory reference intervals applicable during the study period. The biochemical profiles were categorized as follows: primary hypergonadotropic hypogonadism, high FSH and LH with low testosterone; secondary hypogonadism, low or inappropriately normal FSH and LH with low testosterone; primary spermatogenic failure, high FSH with normal LH and testosterone; biochemical profile consistent with partial androgen resistance, normal FSH with elevated LH and testosterone; hyperprolactinemia, elevated prolactin; and normal hormonal profile, FSH, LH, testosterone, and prolactin within their respective reference intervals. The six categories were analyzed as mutually exclusive categories.

Hormone assay methodology

Venous blood samples were collected from 7 a.m. to 10 a.m. for measurement of serum FSH, LH, total testosterone, and prolactin. Hormonal measurements were performed using electrochemiluminescence immunoassay on the Cobas® e 411 analyzer platform (Hitachi High-Technologies Corporation, Tokyo, Japan). The laboratory reference intervals used during the study period were FSH 1.2-15.8 IU/L, LH 1.7-8.6 IU/L, testosterone 249-836 ng/dL, and prolactin 4-15 ng/mL. Internal quality-control materials were analyzed in accordance with the laboratory's routine quality-assurance procedures. Hormonal concentrations were categorized as low, normal, or high using the laboratory reference intervals applicable during the study period.

Ethical considerations

The study was conducted following approval from the Institutional Review Board of Sheikh Zayed Medical College/Sheikh Zayed Hospital (IRB approval number: 163/IRB/SZMC/SZH, dated 26/12/2020).

Statistical analysis

Data were analyzed using IBM SPSS Statistics for Windows, Version 25 (Released 2017; IBM Corp., Armonk, New York). Categorical variables were summarized as frequencies and percentages. Hormonal-profile categories were compared between participants with oligozoospermia and azoospermia using exact tests because of the relatively small number of participants in several categories. The overall distribution of the six mutually exclusive hormonal-profile categories between the two semen-analysis groups was assessed using an exact test for the 2 × 6 contingency table. Exploratory category-specific comparisons were performed using two-sided Fisher's exact tests. A two-sided P-value <0.05 was considered statistically significant. Because the overall and category-specific analyses were based on categorical frequency data, no t-test or F-test statistics were applicable.

## Results

Among the 213 infertile males included in the study, 123 (57.7%) had oligozoospermia and 90 (42.3%) had azoospermia. A normal hormonal profile was observed in 77 (62.6%) oligozoospermic participants and 48 (53.3%) azoospermic participants. Accordingly, one of the defined hormonal abnormalities was present in 46 (37.4%) participants with oligozoospermia and 42 (46.7%) participants with azoospermia.

Among azoospermic males, primary hypergonadotropic hypogonadism was identified in seven (7.8%), secondary hypogonadism in 19 (21.1%), primary spermatogenic failure in nine (10.0%), a biochemical profile categorized as partial androgen resistance in four (4.4%), and hyperprolactinemia in three (3.3%) participants. Among oligozoospermic males, the corresponding frequencies were four (3.3%), 24 (19.5%), 11 (8.9%), five (4.1%), and two (1.6%), respectively.

The overall distribution of the six hormonal-profile categories did not differ significantly between oligozoospermic and azoospermic participants (exact P=0.593). Similarly, exploratory category-specific Fisher's exact tests did not demonstrate statistically significant differences between the two groups (all P>0.05; Tables 1, 2).

**Table 1:** Hormonal profile evaluation among oligozoospermic and azoospermic males (n=213) Overall comparison of the six-category hormonal-profile distribution: exact P=0.593. Category-specific P-values represent exploratory two-sided Fisher's exact tests.

Hormonal profile	Oligozoospermia n=123	Azoospermia n=90	P-value
Primary hypergonadotropic hypogonadism	4 (3.25%)	7 (7.78%)	0.209
Secondary hypogonadism	24 (19.5%)	19 (21.1%)	0.863
Primary spermatogenic failure	11 (8.9%)	9 (10.0%)	0.816
Partial androgen resistance	5 (4.1%)	4 (4.4%)	1.000
Hyperprolactinemia	2 (1.62%)	3 (3.33%)	0.652
Normal hormonal profile	77 (62.6%)	48 (53.3%)	0.205

**Table 2:** Odds ratio (95% CI), azoospermia vs. oligozoospermia ORs are conditional odds ratios comparing azoospermic with oligozoospermic participants; 95% confidence intervals are shown. All confidence intervals included unity.

Hormonal profile	OR (95% CI)
Primary hypergonadotropic hypogonadism	2.50 (0.61–12.02)
Secondary hypogonadism	1.10 (0.53–2.28)
Primary spermatogenic failure	1.13 (0.39–3.16)
Partial androgen resistance	1.10 (0.21–5.26)
Hyperprolactinemia	2.08 (0.23–25.38)
Normal hormonal profile	0.68 (0.38–1.23)

## Discussion

The principal finding of the present study was that hormonal abnormalities were observed in a substantial proportion of infertile men in both semen-analysis groups. Among men with oligozoospermia, 37.4% had one of the defined abnormal hormonal profiles, compared with 46.7% of men with azoospermia. Conversely, a normal profile of FSH, LH, testosterone, and prolactin was observed in 62.6% and 53.3% of the two groups, respectively. However, the overall distribution of hormonal-profile categories did not differ significantly between oligozoospermic and azoospermic participants. Thus, although numerical differences were present for individual categories, these differences should be interpreted descriptively and should not be considered evidence of distinct endocrine distributions between the two groups.

Secondary hypogonadism represented one of the most frequently identified abnormalities in both groups, occurring in 19.5% of oligozoospermic and 21.1% of azoospermic men. This finding emphasizes the relevance of assessing the hypothalamic-pituitary-gonadal axis during the evaluation of male infertility. Hypogonadotropic hypogonadism is characterized by deficient hypothalamic or pituitary stimulation resulting in low testosterone associated with low or inappropriately normal gonadotropin concentrations. Fraietta et al. similarly emphasized the importance of identifying hypothalamic-pituitary dysfunction in infertile men [10].

Primary hypergonadotropic hypogonadism was numerically more frequent among azoospermic than oligozoospermic men (7.8% versus 3.3%), although this difference was not statistically significant. Elevated gonadotropin concentrations in association with reduced testosterone may indicate impaired testicular endocrine function. Similarly, primary spermatogenic failure was identified in 10.0% of azoospermic and 8.9% of oligozoospermic participants. These findings support the role of FSH, LH, and testosterone measurements in differentiating potential testicular from hypothalamic-pituitary causes of impaired spermatogenesis [11,12].

Hyperprolactinemia was relatively uncommon in this study, being observed in 3.3% of azoospermic and 1.6% of oligozoospermic participants. Although the absolute frequency was low, measurement of prolactin may be clinically relevant in appropriately selected patients because hyperprolactinemia can interfere with hypothalamic-pituitary-gonadal function. Previous reports have also identified endocrine abnormalities, including hypogonadotropic hypogonadism and hyperprolactinemia, among potential contributors to male infertility [13,14].

An important finding was that more than half of the participants in both groups had normal concentrations of the four hormones assessed. A normal limited hormonal profile does not exclude male-factor infertility, because spermatogenic impairment may result from genetic, anatomical, obstructive, environmental, metabolic, or idiopathic causes that are not detected by measurement of FSH, LH, testosterone, and prolactin alone. This observation emphasizes that hormonal testing should be interpreted as one component of a comprehensive male-infertility evaluation rather than as an isolated diagnostic approach [11].

The absence of a statistically significant difference in the overall hormonal-profile distribution between oligozoospermic and azoospermic men is also clinically relevant. Semen phenotype alone may not reliably predict a specific endocrine abnormality, and patients within both groups may demonstrate heterogeneous underlying mechanisms. These findings therefore favor individualized evaluation rather than assuming that azoospermia or oligozoospermia corresponds to a particular hormonal pattern [11].

This study has several limitations. First, its cross-sectional design permits description of associations but does not establish causal relationships between hormonal abnormalities and infertility phenotypes. Second, the study was conducted at a single tertiary-care hospital using non-probability consecutive sampling; consequently, referral bias and selection bias are possible, and the findings may not be generalizable to infertile men in the wider community. Third, only FSH, LH, testosterone, and prolactin were evaluated, while other endocrine, genetic, anatomical, metabolic, and environmental causes of infertility were not comprehensively assessed. A fertile control group was also not included.

The study did not distinguish obstructive from non-obstructive azoospermia, although these entities may demonstrate substantially different hormonal characteristics. Some clinically relevant etiological variables, including cryptorchidism, were not separately evaluated. In addition, the hormonal categories used in this study represent biochemical patterns rather than definitive etiological diagnoses, and misclassification may therefore have occurred. Potential confounding variables were not evaluated using multivariable analysis.

Because the study was conducted several years before manuscript revision, complete patient-level raw data and some granular laboratory procedural documentation were no longer available for retrospective verification. Consequently, the statistical discrepancies identified during peer review were corrected using the aggregate frequencies reported in the original results table rather than by reconstructing individual-level observations. The original sample-size calculation worksheet was also unavailable and could not be independently verified. These limitations should be considered when interpreting the findings.

## Conclusions

Hormonal abnormalities were identified in both oligozoospermic and azoospermic infertile men, with abnormal profiles observed in 37.4% and 46.7% of participants, respectively. However, the overall distribution of the evaluated hormonal-profile categories did not differ significantly between the two groups. More than half of the participants in each group had normal FSH, LH, testosterone, and prolactin concentrations, highlighting the heterogeneous and multifactorial nature of male infertility. Hormonal evaluation should therefore be considered as one component of a comprehensive etiological assessment rather than as a stand-alone diagnostic approach. Further prospective studies incorporating broader endocrine, genetic, anatomical, and clinical evaluation are required to confirm these findings.
